# Atypical Response in Metastatic Non-Small Cell Lung Cancer Treated with PD-1/PD-L1 Inhibitors: Radiographic Patterns and Clinical Value of Local Therapy

**DOI:** 10.3390/cancers15010180

**Published:** 2022-12-28

**Authors:** Shanshan Jiang, Jinmeng Zhang, Li Chu, Xiao Chu, Xi Yang, Yida Li, Tiantian Guo, Yue Zhou, Dayu Xu, Jiuang Mao, Zhiqin Zheng, Yulin An, Hua Sun, Huiling Dong, Silai Yu, Ruiting Ye, Jie Hu, Qian Chu, Jianjiao Ni, Zhengfei Zhu

**Affiliations:** 1 Department of Radiation Oncology, Fudan University Shanghai Cancer Center, Shanghai 200032, China; 2 Department of Oncology, Shanghai Medical College, Fudan University, Shanghai 200032, China; 3 Shanghai Clinical Research Center for Radiation Oncology, Shanghai 200032, China; 4 Shanghai Key Laboratory of Radiation Oncology, Shanghai 200032, China; 5 Department of Pulmonary Medicine, Zhongshan Hospital, Fudan University, Shanghai 200032, China; 6 Department of Oncology, Tongji Hospital, Tongji Medical College, Huazhong University of Science and Technology, Wuhan 430000, China

**Keywords:** NSCLC, atypical response, PD-1/PD-L1 inhibitors, local therapy, progression-free survival, overall survival

## Abstract

**Simple Summary:**

The emergence of atypical response (AR) has challenged the process of response evaluation and the subsequent management of non-small cell lung cancer (NSCLC) patients treated with PD-1/PD-L1 inhibitors. We conducted a multicenter retrospective analysis and found that AR was not an uncommon event in patients with metastatic NSCLC treated with the PD1/PD-L1 inhibitor. The median time to AR occurrence was 2.0 months, and patients with ≥3 metastatic organs at baseline were more likely to develop AR. For patients with AR, the common sites of progressive lesions were the lymph nodes and lungs. Furthermore, the majority of patients with AR had only 1–2 progressive tumor lesions, and most of the progressive lesions developed from originally existing tumor sites. Patients with AR had a comparable prognosis to those with a typical response (TR). Proper local therapy targeting progressive tumor lesions while maintaining the PD1/PD-L1 inhibitor may be a feasible treatment selection for patients with AR.

**Abstract:**

Purpose: To explore the clinical characteristics, management, and survival outcomes of advanced NSCLC patients treated with PD-1/PD-L1 inhibitors who presented with an atypical response (AR). Methods: A total of 926 PD-1/PD-L1-inhibitor-treated patients with metastatic NSCLC from three academic centers were retrospectively reviewed. All measurable lesions were evaluated by RECIST version 1.1. Results: Fifty-six (6.1%) patients developed AR. The median time to the occurrence of AR was 2.0 months. Patients with no fewer than 3 metastatic organs at baseline were more prone to develop AR in advanced NSCLC (*p* = 0.038). The common sites of progressive lesions were lymph nodes (33.8%) and lungs (29.7%). The majority (78.2%) of patients with AR had only 1–2 progressive tumor lesions, and most (89.1%) of the progressive lesions developed from originally existing tumor sites. There was no significance in terms of survival between patients with AR and those with typical response (TR). Local therapy was an independent predictor for PFS of patients with AR (*p* = 0.025). Conclusions: AR was not an uncommon event in patients with metastatic NSCLC treated with PD-1/PD-L1 inhibitors, and it had a comparable prognosis to those with TR. Proper local therapy targeting progressive lesions without discontinuing original PD-1/PD-L1 inhibitors may improve patient survival.

## 1. Introduction

The emergence of immune checkpoint inhibitors has resulted in significant progression in the treatment of advanced non-small cell lung cancer (NSCLC) [1,2,3]. Administered as monotherapy or in combination with chemotherapy, PD-1/PD-L1 inhibitors result in improved overall survival (OS) of stage IV NSCLC among untreated and pretreated patients [1,3,4]. However, the radiological evaluation of PD-1/PD-L1 inhibitor-treated NSCLC is challenging due to the occurrence of atypical response (AR), manifesting as simultaneous regression in some tumors and progression in others [5]. The development of AR complicates the evaluation of treatment efficacy and decisions regarding subsequent treatment strategies. Although several versions of imaging-response standards related to the PD1/PD-L1 inhibitor have been explored to shrink the impact of AR patterns with regard to tumor-response evaluation [6,7], real-world clinical descriptions of this immune-related response pattern and treatment strategy in NSCLC remain scarce. Moreover, the prognostic significance of AR is not yet fully understood. Studies reported that the prognosis of patients with AR was superior to those of patients with typical progression but inferior to those with a typical response (TR) [5,8,9,10]. However, these studies had limited sample sizes and were generally insufficient to capture the full picture of AR in advanced NSCLC. For example, the radiographic patterns and clinical management of patients with AR have seldom been investigated.

Local consolidative therapy is an interesting topic for the treatment of oligo-progressive sites, and it has been increasingly provided in clinical practice. A wealth of research has demonstrated the essential role of local cancer treatments in improving survival among NSCLC patients with oligo-progressive or oligometastic disease [11,12,13]. Radiotherapy has immunomodulatory qualities capable of augmenting antitumor immune responses, making the integration of radiotherapy with immunotherapy a new therapeutic option in advanced NSCLC [14,15]. However, few studies have commented on treatment recommendations for patients with AR in NSCLC. Here, a retrospective, multicenter, collaborative study was performed to investigate the clinical characteristics, treatment strategy, and survival results of AR in metastatic NSCLC patients treated with PD1/PD-L1 inhibitor.

## 2. Methods

### 2.1. Patients

In this study, AR was defined as the simultaneous occurrence of an objective response in some tumor lesions and disease progression in others within the PD-1/PD-L1 inhibitor treatment window, as depicted in a previous study [6]; otherwise, it was defined as TR. In the current study, the medical records of advanced NSCLC patients treated with PD1/PD-L1 inhibitor at Fudan University Shanghai Cancer Center, Fudan University Zhongshan Hospital, and Tongji Hospital affiliated with Tongji Medical College of Huazhong University of Science and Technology from May 2018 to January 2022 were reviewed. Two patient groups were selected on the basis of PD1/PD-L1 inhibitor response patterns. One group (TR group) included patients with complete responses (CRs), partial responses (PRs), and durable stable disease (SD, duration > 6 months) according to the response evaluation criteria in solid tumors (RECIST) version 1.1 criterion as previously described [16]. Another group (AR group) included patients who developed AR. All of the included patients were diagnosed as having non-small cell lung cancer at the Department of Pathology in each medical center. Histopathological types were aggregated into squamous-cell carcinoma and non- squamous-cell carcinoma, which included adenocarcinoma, large cell carcinoma, and others. The Dako PD-L1 immunohistochemical 22C3 and 28-8 pharmaDx assays employ tumor proportion score (TPS) to measure PD-L1 expression. TPS cutoffs for low and high expression were 1% and 50%, respectively [17]. The other inclusion criteria included adequate follow-up duration and at least one measurable lesion. Patients with secondary primary tumors, sensitizing driver gene mutations, or those lost to follow-up were excluded from the study. Baseline clinicopathological parameters and treatment details were collected for each patient, including age, sex, pathology, smoking status, ECOG PS score, treatment, and PD-L1 expression. The dates of patients’ disease progression, final follow-up, and death were also recorded. The current study was ethically approved by the three institutional review committees (approval number: 2012228-3).

### 2.2. Follow-Up

Patients were generally followed-up using chest computed tomography (CT), abdominal CT, or ultrasound examination every 6–12 weeks. Brain magnetic resonance imaging (MRI), bone scans, and PET/CT were not mandatory, and they generally performed until indicative symptoms and/or clinical signs were observed. The data were cut-off on 31 January 2022.

### 2.3. Pattern of Response

Considering the potential heterogeneity in response, the full range of radiologic behavior was captured to identify AR at the individual lesion and organ levels. Serial imaging scans of each patient were reviewed by two experienced radiologists. Each tumor lesion with measurable disease was independently evaluated. For patients with AR, the initial time of documentation of AR was recorded. In addition, unidimensional measurements (mm) were used to quantified the absolute size and percent change of the overall tumor burden, which was evaluated in accordance with RECIST version 1.1.

### 2.4. Statistical Analysis

For patients with TR, progression-free survival (PFS) was computed from the beginning of PD1/PD-L1 inhibitor treatment to the initial disease progression; the death of any cause; or, if no progression was observed, censored at the data lock. For patients with AR, PFS was calculated from the initiation of the PD1/PD-L1 inhibitor to the time of the first disease progression after the occurrence of AR (regardless of whether there was initial RECIST-defined disease progression before the occurrence of AR), or death. OS was computed from the beginning of the PD1/PD-L1 inhibitor to death of any cause or, if no death was observed, censored at the time point of the final follow up. The patients’ survival was plotted using the Kaplan–Meier method, and the differences in the variables were estimated by log-rank test. Cox proportional hazard models were used to calculate the hazard ratios (HRs) and 95% confidence intervals (CIs). All of the significant factors with a *p*-value < 0.10 in the univariate analysis were finally included in the multivariate analysis. The propensity score matching (PSM) was performed on the basis of the logic of propensity score and one-to-one nearest neighbor matching to further reduce the potential bias of covariates. The caliper was 0.20 [18], and a two-sided *p* value < 0.05 was defined as statistical significance. All statistical tests were performed on SPSS (version 22.0; SPSS Inc., Chicago, IL, USA). For survival analysis, R software version 4.1.3 (The R Foundation for Statistical Computing Platform) was used for computation.

## 3. Results

### 3.1. Patient’s Characteristics

**Figure 1:**
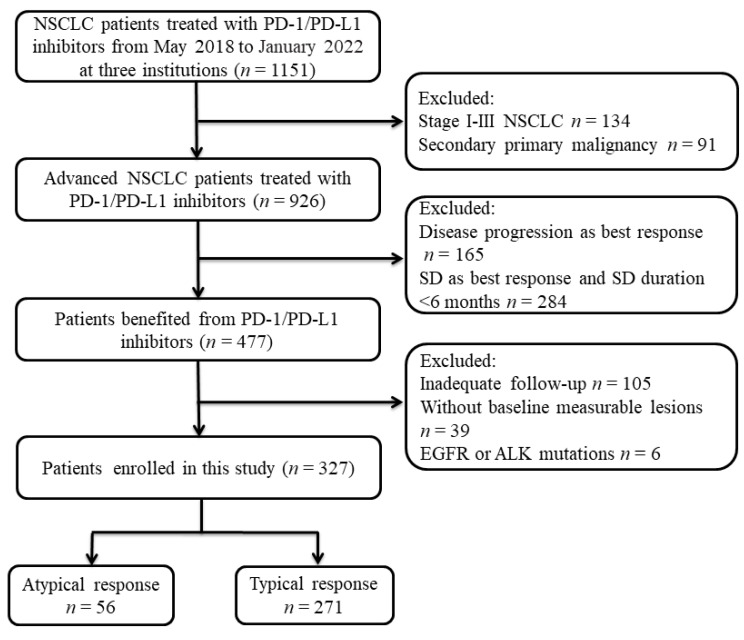
Flowchart of patient enrollment.

A total of 327 of the 926 immune-checkpoint-inhibitor-treated patients with metastatic NSCLC were enrolled, with 271 cases in the TR group and 56 in the AR group. The details of patient enrollment are shown in Figure 1. In the whole population, PD-1/PD-L1 inhibitors were used a as first- and second-line or further-line treatment in 141 (43.1%) and 186 (56.9%) patients, respectively. The majority (68.5%) of the patients received combination treatment, while 31.5% of the patients were treated with PD-1/PD-L1 inhibitor monotherapy. The characteristics of the enrolled patients in the AR and TR groups are displayed in Table 1.

### 3.2. Clinical Features of Atypical Response

**Figure 2:**
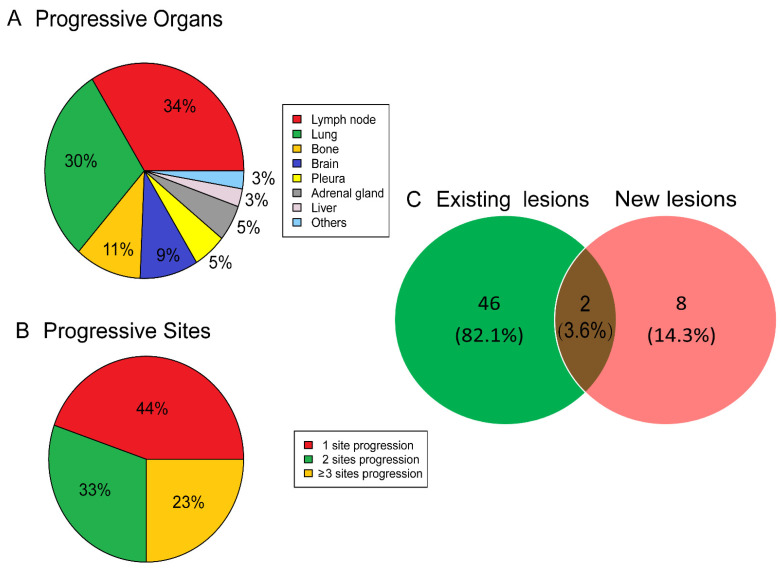
Progressive patterns of AR. Locations (**A**), number (**B**), and originations (**C**) of the progressive tumor lesions in patients with AR.

The frequency of AR in the current case cohort was 6.1% (56/926, 6.1%), and the median time between the beginning of PD1/PD-L1 inhibitor to the documentation of AR was 2.0 (range of 1.0–5.0) months. At the time of the initial documentation of AR, the response patterns per RECIST version 1.1 were determined to be PRs, SD, and disease progression in 12, 35, and 9 patients, respectively. For each patient with AR, the site and number of initial progressive tumor lesions that led to their classification as AR were carefully recorded. The common organs with progressive lesions were lymph nodes (33.8%), lungs (29.7%), bones (10.8%), brain (9.5%), pleura (5.4%), adrenal gland (5.4%), and liver (2.7%, Figure 2A). Meanwhile, 138 progressive tumor lesions were observed in 56 AR patients. Twenty-five (44.6%) patients had only a single progressive lesion, 18 (32.2%) patients had two progressive tumor lesions, and the remaining 13 (23.2%) patients had ≥three progressive tumor lesions (Figure 2B). Among the 56 patients with AR, progressive tumor lesions developed from the originally existing tumor sites in 46 patients (82.1%) and newly emergent tumor lesions leading to the development of AR occurred in eight patients (14.3%); the remaining two (3.6%) patients had progressive tumor lesions at the original sites and the new tumor deposits (Figure 2C).

Univariate analysis revealed that the number of baseline metastatic organs and metastatic sites was related to AR. There was no significance between age, sex, ECOG PS score, smoking status, histology, treatment regimens, treatment lines, PD-L1 expression, and the occurrence of AR. In multivariate analysis, the number of baseline metastatic organs was found to be a significant factor of AR (*p* = 0.038, Table 1), which was corroborated by a previous study on metastatic melanoma [19].

### 3.3. Prognostic Significance of Atypical Response

**Figure 3:**
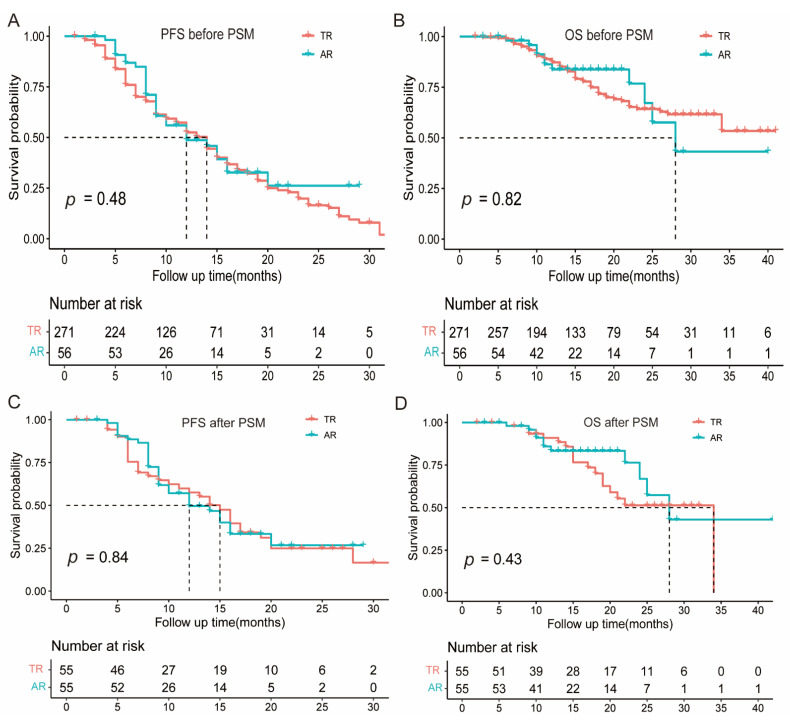
Kaplan–Meier analysis of PFS and OS. Kaplan–Meier plots of PFS (**A**), and OS (**B**) in patients with AR and TR before PSM. Kaplan–Meier plots of PFS (**C**), and OS (**D**) in patients with AR and TR after PSM. AR, atypical response; TR, typical response.

With a median follow-up of 16 (range of 2–52) months, 199 patients developed disease progression per RECIST version 1.1, and 75 patients died. In the whole population (*n* = 327), the median OS and PFS were not reached and 13.0 (95% CI: 12.0–15.0) months, respectively (Figure S1). Furthermore, no significance in PFS (Figure 3A) or OS (Figure 3B) was found between the AR and TR groups. Cox analyses revealed that the response pattern (AR vs. TR) was not associated with PFS (Table S1) and OS (Table S2).

PSM analysis was performed, including four covariates (sex, number of metastatic organs, treatment regimens, and treatment lines), to reduce the potential confounding factors that may exist between patients with AR and TR. The baseline characteristics of the matched cohorts after PSM were well balanced (Table S3), and no significant differences in PFS (Figure 3C) and OS (Figure 3D) were found between patients with AR and TR. The detailed survival outcomes of PSM are shown in Tables S4 and S5, respectively.

### 3.4. The Value of Local Treatment for Atypical Response

**Figure 4:**
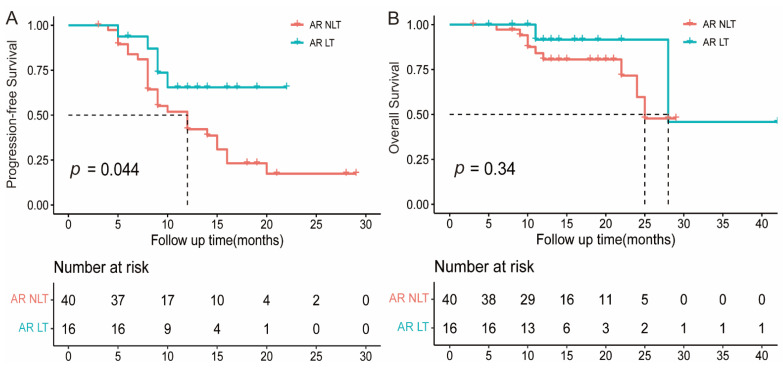
Kaplan–Meier analysis of PFS (**A**) and OS (**B**) in patients with AR who received local therapy or not. AR, atypical response; NLT, non-local therapy; and LT, local therapy.

In this study, all AR patients continued their original PD1/PD-L1 inhibitor, and 16 (28.6%) patients received local therapy targeting the paradoxical progressive lesions after the development of AR, including radiotherapy in 13, surgery in 2, and other local interventions in the remaining 1 patient. The baseline characteristics between those with and without local therapy were generally balanced (Table S6), and local therapy resulted in a notably prolonged PFS (not reached vs. 12.0 months, Figure 4A) and a numerically longer OS (28.0 vs. 25.0 months, Figure 4B). Cox analyses also revealed that local therapy was a significant positive predictor of PFS in the AR group (Table S7).

## 4. Discussion

In the current study, a multicenter retrospective analysis was conducted to investigate the clinical features, prognostic significance, and treatment of patients with stage IV NSCLC with AR receiving immune checkpoint inhibitors. The outcomes of PD1/PD-L1 inhibitor in patients with AR were comparable to those in patients with TR. Moreover, proper local therapy targeting paradoxical progressive lesions may provide survival benefits for patients with AR, which warrants further validation.

The clinical features of AR are not fully understood, and this study provides significant information about the frequency and spatiotemporal patterns of AR in patients with metastatic NSCLC treated with PD1/PD-L1 inhibitor. The frequency of AR was reported to be from 5.0% to 13.0% in PD-1/PD-L1 inhibitor-treated metastatic NSCLC [5,10,20], which was consistent with the current study (6.1%). Previous studies [5,9] reported a median interval to AR of within 3 months, which was similar to the finding of the present study. Hence, the paradoxical progressive disease that developed during the first 2–3 months needed to be interpreted with caution because a non-negligible percentage of patients may actually have AR. In addition, all lesions during the initial radiological examination were evaluated, and patients with more than three metastatic organs or sites were found to be more likely to develop AR. The spatial distribution of progressive lesions in patients with AR, in terms of sites and number, was quite similar to that reported in those with acquired resistance to PD1/PD-L1 inhibitor [5,21,22,23,24,25]. In both clinical scenarios, the common sites of progressive disease often occurred in the lymph nodes and lung, and the most common progressive lesion mainly occurred in the originally existing sites; meanwhile, the majority of patients had no more than three progressive lesions, indicating potential shared mechanisms regarding intra-tumoral heterogeneity and reductant immune evasion pathways in metastatic NSCLC [22,26,27,28]. Accordingly, a previous study reported that acquired resistance to the PD1/PD-L1 inhibitor usually occurred in one or two sites of disease when local treatment and the continuous PD1/PD-L1 inhibitor led to prolonged benefits [22], in line with the results of the current research.

However, the mechanism underlying AR remains unclear. The heterogeneity and differences of intra-tumoral between metastatic sites may contribute to fluctuations in clinical effects (i.e., some tumor lesions shrinkage while others growth). Moreover, it takes some time to activate the immune system, the start of clinical effects may be delayed, and the interaction between the immune system and the tumor may be a long-term process that could possibly contribute to some sites shrinking while other sites are grow or new lesions emerge [20]. PD-L1 expression, as an important biomarker for immune checkpoint inhibitors and an efficient and crucial checkpoint of acquired immune resistance in NSCLC, was found to be different among tumor specimens of different sites in patients with NSCLC [29,30,31], highlighting the magnitude of intra-tumoral immune heterogeneity. A notable detail is that tumor microenvironments, such as T cell recruitment and infiltration, may influence the outcome of PD-1/PD-L1 inhibitors and differ across metastatic organs in patients with tumor [32]. Meanwhile, patients with AR often experienced concomitant increases in some lesions and a decrease in other lesions, indicating that patients with AR could benefit from the PD1/PD-L1 inhibitor and continued PD-1/PD-L1 inhibitor combination treatment beyond the first disease progression on RECIST version 1.1 compared with patients who stopped the PD1/PD-L1 inhibitor and changed anticancer therapy [23,33,34,35]. Therefore, the continuation of the PD1/PD-L1 inhibitor beyond progression may be appropriate for patients with AR in advanced NSCLC.

The value of local therapy for stage IV NSCLC patients in an AR cohort treated with PD-1/PD-L1 inhibitors has seldom been reported. A retrospective study reported that the duration of PD1/PD-L1 inhibitors could be amplified by local therapy, such as radiotherapy, to solitary or oligo-progressive sites in atypical response, especially among those harboring a biologically more indolent cancer type, such as renal cancer [24]. Moreover, surgery or radiotherapy are becoming an integral component in the treatment of oligo-progressive disease in NSCLC [36]. Local consolidative therapy with or without maintenance therapy for patients with three or fewer metastases from NSCLC that did not progress after initial systemic therapy improved progression-free survival and overall survival compared with maintenance therapy alone [13,37]. In the present study, most progressive tumor lesions developed from the originally existing tumor sites in patients with AR. Proper local therapy for progressive lesions in patients with AR could prolong PFS in NSCLC and thus extend the PD1/PD-L1 inhibitor treatment duration, which is generally related to less toxicity and better quality of life than conventional chemotherapy [1]. The current findings regarding the clinical value of local therapy within the PD1/PD-L1 inhibitor window in patients with AR were comparable to those reported in patients with metastatic NSCLC treated with the PD1/PD-L1 inhibitor who developed acquired resistance [22,23,38]. Local therapy, such as radiotherapy, targeting oligo-progressive tumor lesions in patients with AR or acquired resistance may improve patient survival as it could effectively eradicate tumor lesions compromising PD1/PD-L1-inhibitor-resistant subclones and enhance the systemic antitumor immune response through synergistic effects between radiotherapy and immunotherapy [38,39,40]. However, the current work may be the first to report the potential clinical benefit of local therapy for progressive targeted sites or new lesions in patients with AR in advanced NSCLC, thereby warranting future investigation.

This study has some limitations. First, selection bias and unrecognized confounding factors could exist between subgroups, in which survival outcomes were compared. Although potential bias was reduced as much as possibly by using Cox analyses and PSM, imbalanced baseline characteristics could be inevitable. Thus, the findings in the present study need to be validated by prospective randomized trials. Second, as a retrospective study, collecting detailed information about treatment toxicities was difficult, and thus the safety profile of adding local therapy needs to be investigated in further prospective studies.

## 5. Conclusions

In conclusion, AR was not an uncommon event in patients with metastatic NSCLC treated with PD1/PD-L1 inhibitor, and it had a comparable prognosis to those with TR. Proper local therapy targeting progressive tumor lesions while maintaining the PD1/PD-L1 inhibitor may be a feasible treatment selection for patients with AR, thus warranting further investigation.

## Figures and Tables

**Table 1:** Baseline characteristics and logistic regression analysis for predictors of atypical response.

	AR	TR	Univariate Analysis				Multivariate Analysis			
	(*N* = 56)	(*N* = 271)	HR	95% CI		*p*	HR	95% CI		*p*
Age, years										
≤62	25 (44.6%)	103 (38.0%)	1							
>62	31 (55.4%)	168 (62.0%)	0.760	0.425	1.359	0.355				
Gender										
Male	45 (80.4%)	217 (80.1%)	1							
Female	11 (19.6%)	54 (19.9%)	0.982	0.476	2.025	0.961				
ECOG PS Score										
0–1	52 (92.9%)	252 (19.0%)	1							
2	4 (7.1%)	19 (81.0%)	1.020	0.333	3.123	0.972				
Smoking status										
Ever	26 (46.4%)	152 (56.1%)	1							
Never	30 (53.6%)	119 (43.9%)	0.903	0.507	1.609	0.73				
Histology										
Squamous-cell Carcinoma	14 (25.0%)	105 (38.7%)	1				1			
Non-squamous-cell Carcinoma	42 (75.0%)	166 (61.3%)	1.898	0.988	3.644	0.054	1.853	0.946	3.628	0.072
NO. of metastatic organs										
≤3	45 (80.4%)	255 (94.1%)	1				1			
>3	11 (19.6%)	16 (5.9%)	3.896	1.698	8.939	0.001	2.708	1.056	6.943	0.038
NO. of metastatic sites										
≤3	29 (51.8%)	181 (66.8%)	1				1			
>3	27 (48.2%)	90 (33.2%)	1.872	1.046	3.351	0.035	1.425	0.731	2.777	0.298
Treatment regimens										
ICI alone	23 (41.1%)	80 (29.5%)	1				1			
ICI combination	33 (58.9%)	191 (70.5%)	0.601	0.332	1.087	0.092	0.575	0.309	1.071	0.081
Treatment lines										
1st	27 (48.2%)	114 (42.1%)	1							
≥2nd	29 (51.8%)	157 (57.9%)	0.780	0.438	1.389	0.398				
PD-L1 expression, %										
<1	4 (7.1%)	18 (6.6%)	1							
1–49	6 (10.7%)	40 (14.8%)	0.675	0.169	2.689	0.577				
≥50	13 (23.2%)	48 (17.7%)	1.219	0.351	4.231	0.755				
Unknown	33 (59.0%)	165 (60.9%)	0.900	0.286	2.831	0.857				

Abbreviations: ICI, immune checkpoint inhibitors; ECOG PS, eastern cooperative oncology group performance status; AR, atypical response; and TR, typical response.

## Data Availability

The data that support the findings of this study are available from the corresponding author upon request.

## Supplementary Materials

The following supporting information can be downloaded at https://www.mdpi.com/article/10.3390/cancers15010180/s1, Table S1. Cox analyses for progression-free survival in the whole population; Table S2. Cox analyses for overall survival in the whole population; Table S3. Characteristics of patients in propensity-score matched cohort; Table S4. Tumor response and survival analysis in the whole population; Table S5. Survival analysis in propensity score matched cohorts; Table S6. Characteristics of patients with atypical response receiving local therapy or not; Table S7. Cox analyses for progression-free survival in patients with atypical response; and Figure S1. Kaplan–Meier analysis of PFS (A) and OS (B) in the whole population.
