# Supplementary material for: Atypical Response in Metastatic Non-Small Cell Lung Cancer Treated with PD-1/PD-L1 Inhibitors: Radiographic Patterns and Clinical Value of Local Therapy
Source: Cancers (Basel). 2022 Dec 28;15(1):180. doi: 10.3390/cancers15010180 (PMC9818210; doi:10.3390/cancers15010180)
Supplement: Supplementary file 1 [file cancers-15-00180-s001.zip › Supplementary tables.pdf]

Table S1. Cox analyses for progression-free survival in the whole population

	Univariate analysis				Multivariate analysis			
	HR	95% CI		P value	HR	95% CI		P value
Age (≤62y vs >62 y)	1.008	0.977	1.039	0.619				
Gender (Male vs Female)	2.303	1.320	4.018	0.003	2.049	1.039	4.039	0.038
ECOG PS score (0-1 vs 2)	1.664	0.514	5.389	0.396				
Smoking status (Never vs Ever)	0.609	0.366	1.014	0.057	0.834	0.448	1.555	0.569
Histology (Squamous vs Non-squamous)	0.927	0.510	1.685	0.804				
NO. of metastatic organs (>3 vs ≤3)	0.943	0.490	1.813	0.860				
NO. of metastatic sites (>3 vs ≤3)	1.283	0.776	2.123	0.332				
Treatment regimens (ICI alone vs ICI combination)	1.289	0.769	2.162	0.336				
Treatment lines (1st vs $\geq 2^{nd}$)	1.001	0.596	1.681	0.996				
PD-L1 expression, %				0.582				
1-49 vs <1	1.275	0.370	4.396	0.701				
≥50 vs <1	0.662	0.211	2.085	0.481				
Unknown vs <1	0.939	0.335	2.634	0.906				
Response pattern (TR vs AR)	0.823	0.498	1.361	0.448				

Abbreviations: ECOG PS, Eastern Cooperative Oncology Group Performance Status. TR, typical response; AR, atypical response.

Table S2. Cox analyses for overall survival in the whole population

	Univariate analysis			
	HR	95% CI		P value
Age (≤62 y vs >62 y)	0.992	0.950	1.037	0.734
Gender (Male vs Female)	1.048	0.426	2.581	0.918
ECOG PS score (0-1 vs 2)	0.046	0.000	91.526	0.426
Smoking status (Never vs Ever)	0.693	0.329	1.462	0.336
Histology (squamous vs non-squamous)	0.955	0.388	2.348	0.920
NO. of metastatic organs (>3 vs ≤3)	0.935	0.374	2.336	0.885
NO. of metastatic sites (>3 vs ≤3)	2.050	0.951	4.417	0.067
Treatment regimens (ICI alone vs ICI combination)	1.809	0.851	3.846	0.123
Treatment lines (1st vs $\geq 2^{nd}$)	1.064	0.492	2.301	0.874
PD-L1 expression, %				0.515
1-49 vs <1	0.732	0.147	3.635	0.703
≥50 vs <1	0.356	0.083	1.528	0.165
Unknown vs <1	0.495	0.145	1.697	0.263
Response pattern (TR vs AR)	0.702	0.329	1.496	0.359

Abbreviations: ECOG PS, Eastern Cooperative Oncology Group Performance Status. TR, typical response; AR, atypical response.

Table S3. Characteristics of patients in propensity-score matched cohort

	Atypical Response (N=55)	Typical Response (N=55)	P value
Age (years)			1.000
Mean (SD)	60.6 (8.97)	61.5 (8.64)	
Gender			0.818
Male	44 (80.0%)	42 (76.4%)	
Female	11 (20.0%)	13 (23.6%)	
ECOG PS score			1.000
0-1	52 (94.5%)	51 (92.7%)	
2	3 (5.5%)	4 (7.3%)	
Smoking status			0.849
Ever	27 (49.1%)	29 (52.7%)	
Never	28 (50.9%)	26 (47.3%)	
Histology			0.823
Squamous-cell Carcinoma	12 (21.8%)	14 (25.5%)	
Non-Squamous-cell Carcinoma	43 (78.2%)	41 (74.5%)	
NO. of metastatic organs			0.812
≤3	43 (78.2%)	45 (81.8%)	
>3	12 (21.8%)	10 (18.2%)	
NO. of metastatic sites			1.000
≤3	28 (50.9%)	29 (52.7%)	
>3	27 (49.1%)	26 (47.3%)	
Treatment regimens			0.694
ICI alone	19 (34.5%)	22 (40.0%)	
ICI combination	36 (65.5%)	33 (60.0%)	
Treatment lines			0.702
1st	24 (43.6%)	27 (49.1%)	
≥2nd	31 (56.4%)	28 (50.9%)	
PD-L1 expression, %			0.854

<1	2 (3.6%)	4 (7.3%)
1-49	7 (12.7%)	6 (10.9%)
≥50	14 (25.5%)	13 (23.6%)
unknown	32 (58.2%)	32 (58.2%)

Abbreviations: ICI, immune checkpoint inhibitors; ECOG PS, Eastern Cooperative Oncology Group Performance Status; SD, standard deviation.

Table S4. Tumor response and survival analysis in the whole population

Response/Survival	Atypical response (N=56)	Typical response (N=271)	All patients (N=327)
BOR, No. (%)			
Confirmed CRs	0(0.0)	1(0.4)	1(0.3)
Confirmed PRs	30(53.6)	201(74.2)	231(70.6)
SD	26(46.4)	69(25.5)	95(29.1)
PFS, median (95% CI), months	12(9-20)	10(9-12)	11(9-12)
[1]PFS at 6 months, n (%)	46(86.8)	195(72.5)	241(75.0)
[1]PFS at 12 months, n (%)	23(48.1)	82(42.2)	105(43.2)
[1]PFS at 18 months, n (%)	8(32.3)	31(29.2)	39(29.8)
OS, median (95% CI), months	28(24-NR)	-	-
[2]OS at 6 months, n (%)	51(98.0)	250(98.8)	301(98.7)
[2]OS at 12 months, n (%)	34(83.8)	167(87.3)	201(86.7)
[2]OS at 18 months, n (%)	19(76.8)	93(71.7)	112(73.5)

Abbreviations: BOR, best overall response; CRs, complete responses; PRs, partial responses; SD, stable disease; PFS, progression-free survival; OS, overall survival; CI, confidence interval; NR, not reached.

[1]PFS rate was defined as the probability of a patient remaining progression free and alive up to 6, 12,18 months, respectively.

[2]OS rate was defined as the probability of a patient remaining alive up to 6, 12, 18 months, respectively.

Table S5. Survival analysis in propensity score matched cohorts

	Atypical response (N=55)	Typical response (N=55)	All patients (N=110)
PFS, median (95% CI), months	12(9-NR)	10(8-17)	12(9-16)
[1]PFS at 6 months, n (%)	46(88.6)	41(71.6)	87(80.3)
[1]PFS at 12 months, n (%)	23(49.6)	18(42.0)	41(45.9)
[1]PFS at 18 months, n (%)	9(33.0)	9(30.8)	18(32.1)
OS, median (95% CI), months	34(19-NR)	28(24-NR)	34(22-NR)
[2]OS at 6 months, n (%)	50(98.0)	49(98.0)	99(98.0)
[2]OS at 12 months, n (%)	33(83.4)	37(88.5)	70(87.2)
[2]OS at 18 months, n (%)	19(76.4)	21(70.0)	40(75.6)

Abbreviations: PFS, progression-free survival; OS, overall survival; CI, confidence interval; NR, not reached.

[1]PFS rate was defined as the probability of a patient remaining progression free and alive up to 6, 12,18 months, respectively.

[2]OS rate was defined as the probability of a patient remaining alive up to 6, 12, 18 months, respectively.

Table S6. Characteristics of patients with atypical response receiving local therapy or not

	With local therapy (N=16)	Without local therapy (N=40)	P value
Age [mean (range)] years	59.5 (9.83)	62.3 (8.01)	0.326
Gender			0.632*
Male	14 (87.5%)	31 (77.5%)	
Female	2 (12.5%)	9 (22.5%)	
ECOG PS score			0.570*
0-1	14(87.5%)	38(95.0%)	
2	2(12.5%)	2(5.0%)	
Smoking status			0.966
Ever	8 (50.0%)	18 (45.0%)	
Never	8 (50.0%)	22 (55.0%)	
Histology			0.733
Squamous-cell Carcinoma	5 (31.3%)	9 (22.5%)	
Non-Squamous-cell Carcinoma	11 (68.8%)	31 (77.5%)	
NO. of metastatic organs			0.312
≤3	11 (68.8%)	34 (85.0%)	
>3	5 (31.3%)	6 (15.0%)	
NO. of metastatic sites			1.00
≤3	8 (50.0%)	21 (52.5%)	
>3	8 (50.0%)	19 (47.5%)	
Treatment regimens			1.000
ICI alone	7 (43.8%)	16 (40.0%)	
ICI combination	9 (56.3%)	24 (60.0%)	
Treatment lines			0.642
1st	9 (56.3%)	18 (45.0%)	
≥2nd	7 (43.8%)	22 (55.0%)	
PD-L1 expression, %			0.390*

<1	0 (0%)	4 (10.0%)
1-49	3 (18.8%)	3 (7.5%)
≥50	4 (25.0%)	9 (22.5%)
Unknown	9 (56.3%)	24 (60.0%)

Abbreviations: * Fisher's exact method. ICI, immune checkpoint inhibitors; ECOG PS, Eastern Cooperative Oncology Group Performance Status.

Table S7. Cox analyses for progression-free survival in patients with atypical response

	Univariate analysis				Multivariate analysis			
	HR	95% CI		P value	HR	95% CI		P value
Age (≤62y vs >62 y)	1.211	0.583	2.516	0.607				
Gender (Male vs Female)	1.667	0.743	3.738	0.215				
ECOG PS score (2 vs 0-1)	7.924	2.138	29.369	0.002	6.014	1.613	22.425	0.008
Smoking status (Never vs Ever)	1.023	0.503	2.080	0.949				
Histology (Squamous vs Non-squamous)	0.699	0.320	1.524	0.368				
NO. of metastatic organs (>3 vs ≤3)	0.859	0.423	1.744	0.674				
NO. of metastatic sites (>3 vs ≤3)	0.854	0.326	2.236	0.748				
Treatment regimens (ICI alone vs ICI combination)	1.417	0.688	2.919	0.344				
Treatment lines (1st vs ≥2nd)	0.770	0.372	1.594	0.481				
PD-L1 expression, %				0.756				
1-49 vs <1	0.703	0.140	3.545	0.670				
≥50 vs <1	0.454	0.106	1.935	0.286				
Unknown vs <1	0.628	0.185	2.139	0.457				
Local therapy (No vs Yes)	4.322	1.313	14.229	0.016	3.947	1.190	13.086	0.025

Abbreviations: ECOG PS, Eastern Cooperative Oncology Group Performance Status.
