# Supplementary figures and images for: Atypical Response in Metastatic Non-Small Cell Lung Cancer Treated with PD-1/PD-L1 Inhibitors: Radiographic Patterns and Clinical Value of Local Therapy
Source: Cancers (Basel). 2022 Dec 28;15(1):180. doi: 10.3390/cancers15010180 (PMC9818210; doi:10.3390/cancers15010180)

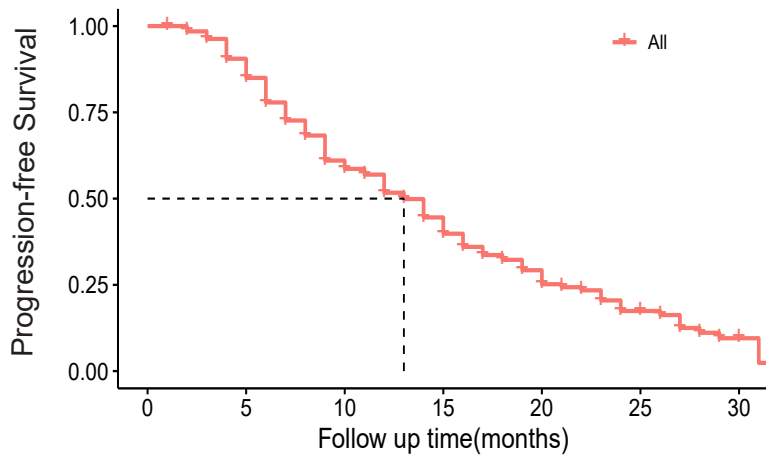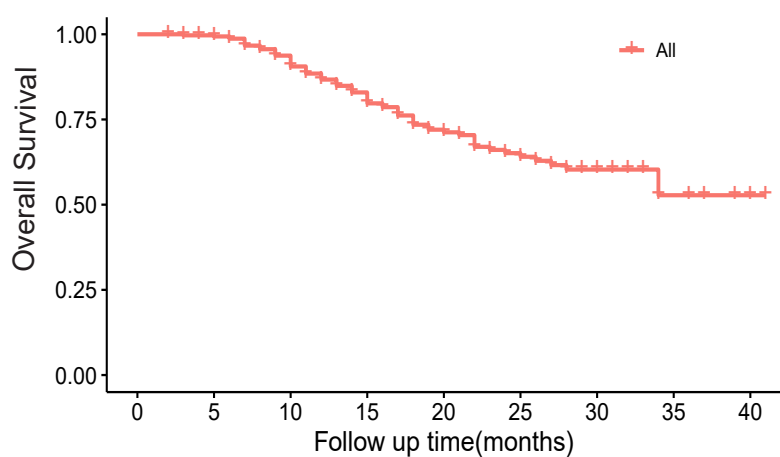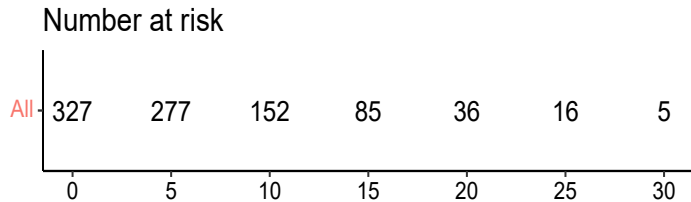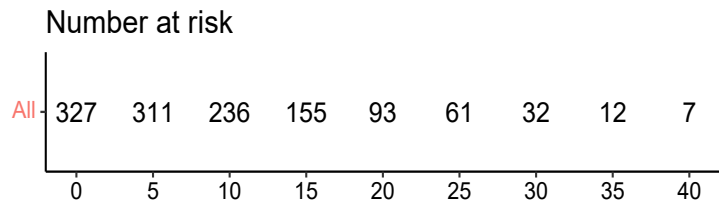

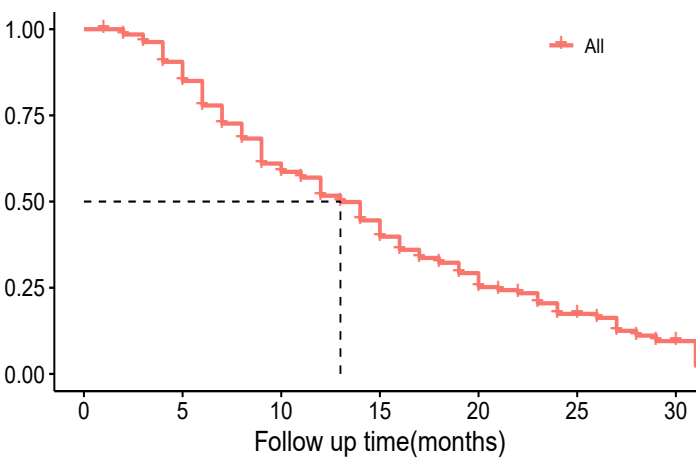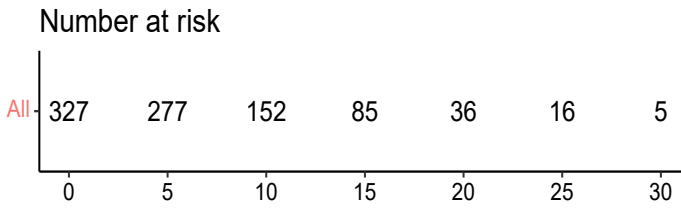

Supplement: Supplementary file 1 [file cancers-15-00180-s001.zip › Figure S1.pdf]
